# A Protocol for the Cryopreservation of Human Intestinal Mucosal Biopsies Compatible With Single-Cell Transcriptomics and *Ex Vivo* Studies

**DOI:** 10.3389/fphys.2022.878389

**Published:** 2022-05-05

**Authors:** Alison McRae, Maria Laura Ricardo-Silgado, Yuanhang Liu, Gerardo Calderon, Daniel Gonzalez-Izundegui, Fariborz Rakhshan Rohakhtar, Vernadette Simon, Ying Li, Andres Acosta

**Affiliations:** ^1^ Precision Medicine for Obesity Program, Department of Gastroenterology and Hepatology, Mayo Clinic, Rochester, MN, United States; ^2^ Division of Biomedical Statistics and Informatics, Mayo Clinic, Rochester, MN, United States; ^3^ Center for Individualized Medicine (CIM), Mayo Clinic, Rochester, MN, United States

**Keywords:** gastrointestinal tract, intestinal biopsy, cryopreservation, FACS-Seq, single-cell transcriptomics, endoscopy, gut epithelium, human primary culture

## Abstract

The heterogeneity of the human intestinal epithelium has hindered the understanding of the pathophysiology of distinct specialized cell types on a single-cell basis in disease states. Described here is a workflow for the cryopreservation of endoscopically obtained human intestinal mucosal biopsies, subsequent preparation of this tissue to yield highly viable fluorescence-activated cell sorting (FACS)isolated human intestinal epithelial cell (IEC) single-cell suspensions compatible with successful library preparation and deep single-cell RNA sequencing (scRNAseq). We validated this protocol in deep scRNAseq of 59,653 intestinal cells in 10 human participants. Furthermore, primary intestinal cultures were successfully generated from cryopreserved tissue, capable of surviving in short-term culture and suitable for physiological assays studying gut peptide secretion from rare hormone-producing enteroendocrine cells in humans. This study offers an accessible avenue for single-cell transcriptomics and *ex vivo* studies from cryopreserved intestinal mucosal biopsies. These techniques may be used in the future to dissect and define novel aberrations to the intestinal ecosystem that lead to the development and progression of disease states in humans, even in rare IEC populations.

## Introduction

The human intestinal epithelium forms a luminal surface to the external environment and represents an extensively dynamic tissue responsible for a diverse array of critical functions. In addition to its major role in food digestion and absorption of nutrients, the intestinal epithelium also plays major roles in protection from microbial infections, coordination of immune responses, and regulation of food intake, intestinal motility, and intestinal homeostasis. Reflective of this diversified range of functions, the intestinal epithelium is profoundly heterogenous in its cellular architecture and is composed of a number of morphologically and physiologically distinct epithelial subtypes, each uniquely specialized to coordinate and effectuate the wide-ranging operations critical to this tissue. Traditional classifications of differentiated intestinal epithelial cell (IEC) subtypes include: nutrient-absorbing enterocytes, mucin-secreting goblet cells, antimicrobial Paneth cells, immune-related tuft cells, hormone-secreting enteroendocrine cells (EECs), and antigen-sampling Microfold cells (M cells), as well as undifferentiated stem cells.

Recent advances in the performance and availability of Next Generation Sequencing (NGS), particularity in single-cell NGS (scNGS) platforms such as single-cell RNA-Sequencing (scRNASeq), have dramatically expanded our understanding of the full diversity encompassing the intestinal epithelium (Grun et al., 2015; Barriga et al., 2017; Haber et al., 2017; Yan et al., 2017; Norkin et al., 2020). As a result, contemporary studies utilizing these new technologies have now demonstrated that within classical subtypes even further heterogeneity and specialization occurs than previously thought, suggesting the development of a classification with additional subtypes may now be necessary (Allaire et al., 2018).

Dysfunction of the gut epithelium has long been associated with a number of both intestinal and systemic disease including: Obesity, metabolic syndrome, Type 2 Diabetes (T2D), irritable bowel syndrome (IBS), inflammatory bowel disease (IBD), microbial infections, cancers, among others (Raybould, 2012; Van Der Heijden and Vermeulen, 2019; Barbara et al., 2021; Riedel et al., 2021). The heterogeneity of the intestinal epithelium has hindered our ability to understand the pathophysiology of distinct specialized cell types on a single-cell basis in human disease, especially in rare subtypes. One example are studies of hormone secreting EECs and their contribution to obesity and diabetes. Although a relatively rare subset of the GI epithelium (<1%), collectively, EECs constitute the largest endocrine system in the body, and play a major role in the regulation of metabolic homeostasis through the release of appetite-regulating hormones (Gribble and Reimann, 2019). Not only are EECs rare in any human sample due to their sparsely dispersed nature among other cell types within the GI tract, EECs are also exceptionally challenging to isolate, limiting our ability to enrich populations for direct study from human tissue. Consequentially our knowledge of the EECs role in the pathogenesis of human obesity remains largely incomplete. However, in addition to broadening insight of the contributions of specialized subtypes of IECs to the homeostasis and functioning of the intestine in health, the progression of NGS technologies to a single-cell resolution has now provided a powerful opportunity for the study of human intestinal tissue to dissect and define novel aberrations to the intestinal ecosystem that lead to the development and progression of disease states in humans, even in rare IEC populations like EECs (Van Der Flier et al., 2009; Parikh et al., 2019; Beumer et al., 2020). Such analyses performed with a sufficient number of participants in case-controlled and interventional studies have the potential to identify novel aberrant mechanistic pathways and distinct pathophysiological characteristics of human IECs that define disease states. Moreover, these studies may allow for the development of improved therapeutic approaches and for discovery of pharmacological targets.

While NGS techniques like scRNASeq represent an extraordinary opportunity to characterize intestinal diseases, studies attempting to perform a transcriptomic analysis of human intestinal tissue need to navigate multiple challenges related to tissue collection and processing, that are common to obtaining various fresh specimens (Rao et al., 2018; Rao et al., 2020). First, clinical research sites must obtain tissue from specific patients for research analysis, which is frequently collected from surgical specimens largely inaccessible to many research institutions. Furthermore, as current scRNAseq methods, including the Chromium 10X genomics platform used in this study, require intact, viable cells for analysis, live tissue is needed. A multitude of intricate technical variables throughout the process of preparing fresh tissue that culminates in analysis with single-cell transcriptomics also renders samples prepared in multiple batches problematic due to technical batch variation, and thus severely limits the number of fresh samples that can be obtained, processed, and analyzed in a single run (Leek et al., 2010; Lahnemann et al., 2020). Fresh tissue specimens can be cryopreserved as intact tissue upon collection using methods similar to those used to cryopreserve viable cells. This technique has recently been employed to overcome the above challenges with some NGS platforms. Current protocols preforming scRNAseq from cryopreserved specimens in kidney, have reported minimal (<5%) transcriptomic alterations in transcriptomes of preserved tissue compared to fresh tissue (Rao et al., 2018; Rao et al., 2020).

In order to expand the accessibility of scRNASeq to larger studies from multiple sites studying GI disease there is a critical need to establish workflows that resolve the methodological limitations to current studies of native human IECs with single-cell analyses, mainly due to acquiring and using fresh tissue samples analyzed in multiple batches. Currently there are no scRNAseq studies utilizing cryopreserved human intestinal biopsy tissue reporting high cell viability and RNA integrity, performed in a single batch.

In the current study, we establish a methodology for the cryopreservation of human intestinal mucosal biopsies and subsequent isolation with fluorescence-activated cell sorting (FACS) coupled with deep transcriptomic analysis of isolated cells using scRNAseq. Collectively, our workflow allowed for the usage of cryopreserved mucosal biopsies, to be FACS-enriched, and further subjected to deep single-cell analysis in a single batch.

## Materials and Methods

### Participants

The study was approved by the Mayo Clinic Institutional Review Board, and all participants gave written informed consent following thorough explanation of the study details. Women of childbearing potential had a negative pregnancy test within 48 h prior to testing. We included men and women, age 18–65, having a stable weight for the previous 3 months. We excluded patients with recent use of weight loss medications (<6 months), history of abdominal GI surgery other than appendectomy, pregnancy, uncontrolled systemic disease, or medications that might interfere with motility, appetite or absorption.

## FACS-Isolation of Cells Originating From Human Cryopreserved Endoscopic Colon Biopsies and Mouse Colon

### Human Tissue Collection

Participants reported to the Mayo Clinic Clinical Research Trials Unit after an 8-h fasting period, and underwent an unsedated flexible sigmoidoscopy subsequent to receiving a tap water enema. During the flexible sigmoidoscopy, 13 mucosal biopsies were obtained from the left colon using a single-use Radial JawTM four Jumbo Biopsy Forceps (M00513361, Boston Scientific). Biopsies were placed in 1 ml chilled HypoThermosol FRS preservation solution (101102, BioLife Solutions), and transferred on ice to the processing laboratory where they were received for immediate handling within 15 min of collection.

### Mouse Tissue Collection

All studies were approved by the Institutional Animal Care and Use Committee at Mayo Clinic. Healthy C57BL/6J mice (*n* = 8) were euthanized under isoflurane inhalation in a sealed chamber of anesthesiology system, followed by cervical dislocation. Tissue samples from the colon (approximately 5 mm) were collected from the distal section of the colon 1.5 cm proximal to the rectum. The muscularis and submucosal layers of the mouse intestine were stripped and discarded, and colonic sections were immediately flushed with cold Phosphate-buffered saline (PBS, 1X).

### Cryopreservation

Human colonic biopsies and mouse colonic sections were washed with cold sterile-PBS, placed in a cryogenic vial (5,000–1,020, Thermo Scientific) containing 1 ml sterile CryoStor^®^ CS10 cryopreservation media (07930, StemCell Technologies), and incubated for 10 min at room temperature. Cryogenic vials containing tissue were placed in a Mr. Frosty Freezing Container (51000001, Thermo Scientific) to control the cooling rate of samples (-1°C/min) and stored at -80°C. After 24-h, samples were transferred to a dewar containing liquid nitrogen, and stored until further use.

### FACS-Isolation

On the day of the experiment, cryopreserved biopsies (*n* = 13 participants) or mouse tissue (*n* = 4 mice) was removed from liquid nitrogen storage and subjected to a rapid thaw by submerging tubes in a 37°C water bath for 5 min. The cryopreservation media was removed from the sample and replaced with 1 ml of pre-warmed RPMI medium (25–506, Genesee Scientific) supplemented with 10% Fetal Bovine Serum (FBS) (v/v) for 15 min at room temperature. Biopsies were washed with cold sterile-PBS and transferred to a low-binding RNase-free microcentrifuge tube (AM12450, ThermoFisher Scientific). Thawed biopsies and biopsies freshly collected the day of the experiment (*n* = 5 patients, 8–13 biopsies each; *n* = 4 mice collected fresh) were minced with sterile surgical scissors and washed twice with Hanks’ Balanced Salt Solution formulated without calcium and magnesium (HBSS –Ca^2+^, Mg^2+^; 14170112, Gibco). Tissue was further dissociated by enzymatic collagenase XI digestion (1.25 mg/ml in 1X HBSS; 1 ml human biopsies, 5 ml mouse colon) for 30 min in a water bath (37°C) with gentle agitation. Approximately 15 min into the enzymatic tissue digestion, samples were subject to gentle mechanical digestion by slowly pipetting 10 times with a 1,000 µl pipette tip, and then placed back into the water bath for the remaining 15 min.

Immediately following digestion, a volume of FBS was added to bring the total concentration in the suspension to 5% (v/v). Tubes were placed on ice, and gently mixed with a pipet. The suspensions were centrifuged at 100 × g 4°C for 30 s to allow undigested mucosa to settle the bottom of the tube. The supernatant fraction was passed through a 70 μm cell strainer and centrifuged at 300 × g 4°C for 5 min. Supernatant fraction was discarded, and the pellet was resuspended in 1xPBS (RNase-free), 2 mM EDTA (RNase-free), and 2% (w/v) Bovine Serum Albumin (BSA) (FACS buffer). The resulting single cell suspensions were stained with the Membrane Permeability Dead Cell Apoptosis Kit (V35123, Invitrogen) with PO-PRO-1 (1:500; early apoptosis marker) and 7-AAD (1:1,000; viability), then incubated in the dark for 20 min on ice and passed through a 30 μm cell strainer just prior to analysis and sorting using a BD FACSAria II cell sorter.

In order to set appropriate gates for sorting, 50,000 events for each unstained, and Fluorescence Minus One (FMO) controls were run. All samples were subjected to the same gating strategy to isolate single, live, non-apoptotic cells. Cell yield and sample composition was further assessed using FlowJo (FlowJo v10.6, FlowJo, LLC). A QC analysis was completed on human FACS-isolated cells 1 h post-sort, by the Mayo Clinic Medical Genome Facility using a Vi-Cell XR to evaluate quality and compatibility of cells with the Chromium 10X Genomics single cell RNA-Sequencing platform.

For further analysis, cells were either used for single cell transcriptomics (below), RNA integrity analysis, or cultured for 24–72 h. Total RNA was extracted from cells using the RNeasy Plus Micro Kit (Cat#74034, Qiagen), and resulting RNA concentration, purity (A260/A280), and RNA integrity number (RIN) was determined using a The Agilent 2,100 Bioanalyzer System. For subsequent culture, isolated cells were washed with 1 ml sterile RNase free PBS, and resuspended in 1 ml culture medium (DMEM Glutamax, 4.5 g/L glucose, Cat#10566024, Gibco; supplemented with 10% (v/v) FBS, 100 units/mL penicillin and 0.1 mg/ml streptomycin). Aliquots (250 μl) were plated into 48-well plates coated with 4 mg/ml Matrigel, and primary cultures were incubated at 37°C in 5% CO^2^.

## Single-Cell RNA-Sequencing

### Tissue Preparation and Single-Cell Transcriptomics

We studied 10 participants [(mean ± SEM): age 39 ± 3.2 years old, BMI 30.2 ± 2.1 kg/m2, 80% females]. All samples were run and sequenced on the same day and instance to avoid detrimental data divergences from batch variations. Thirteen mucosal biopsies originating from the left colon of participants were collected, cryopreserved, and prepared for scRNAseq as described above in “FACS-isolation of cells originating from human cryopreserved endoscopic colon biopsies”. For each participant sample, 600,000 viable, non-apoptotic cells were FACS-isolated into 2 ml of PBS containing 2% (w/v) BSA. Cells were then washed with 1 ml sterile RNase free PBS, and filtered with a 30 μm cell strainer. We performed all steps following the 10X protocol, with a targeted cell capture of 10,000 single cells. We used the Chromium Single Cell 3′ Library & Gel Bead Kit v2 (10X Genomics). In short, all samples and reagents were prepared and loaded into the chip. Then, we ran the Chromium Controller for droplet generation. Reverse transcription was conducted in the droplets. We recovered cDNA through demulsification and bead purification. Pre-amplified cDNA was further subjected to library preparation. Libraries were sequenced on an Illumina Hiseq 4,000 for 100 paired-end runs at 1 sample over 2 lanes.

### scRNAseq Data Analysis

We used 10X Genomics Cellranger Single Cell Software Suite (v3.0.0) to generate FASTQ files, perform alignment to hg38 reference genome, filtering, barcode counting and UMI counting. For subsequent analysis, we followed the integrated analysis workflow in the Seurat package (v3.1) (https://satijalab.org/seurat/v3.1/integration.html). Genes expressed in fewer than 3 cells, cells that expressed fewer than 200 genes and >40% mitochondria genes were excluded for downstream analysis in each sample. Each dataset was normalized using log normalization and scaled for each gene across all cells. All datasets were integrated, scaled, and clustered on the low-dimensional space. Resolution parameter for Seurat was set to 0.3 for all data integrations. Enriched gene markers in each cluster conserved across two conditions were identified with fold change larger than 2, adjusted p-value smaller than 0.05 in both conditions. All clustering and statistical analysis was performed in R (v 3.5.2). Pathway analysis was performed based on Gene Set Enrichment Analysis (GSEA) (Subramanian et al., 2005)and gene sets including ChEA (Lachmann et al., 2010), KEGG pathway (Kanehisa et al., 2016)and GO Biological processes (Ashburner et al., 2000).

## Primary Intestinal Culture Studies

### Generation of Primary Cultures from Human Colonic Biopsies and Mouse Colon

Human colonic biopsies were collected, cryopreserved, and thawed as described above. Thawed biopsies were rinsed with PBS, minced with surgical scissors, and digested with 10 ml collagenase XI (1.25 mg/ml) in PBS at 37°C for 30 min with gentle rotation. Supernatant fractions from each digest were centrifuged twice at 100 g for 5 min to pellet crypts, and resuspended in 8 ml culture medium (DMEM Glutamax, 4.5 g/L glucose, Cat#10566024, Gibco; supplemented with 10% (v/v) FBS, 100 units/mL penicillin and 0.1 mg/ml streptomycin). Aliquots (250 μl) were plated into 48-well plates coated with 4 mg/ml Matrigel, and primary cultures were incubated at 37°C in 5% CO_2_.

### Gut Hormone Secretion Studies

Approximately 12 h after plating cells, media was replaced with minimal DMEM (Cat# A14430, Gibco) supplemented with 1% FBS (v/v) for 2 h to starve cells. Media was then replaced with PBS-0.5% BSA (w/v, control) with or without test compounds: Conjugated Bile Acids (1mM; CBAS, 35% TCA, 21% GCA, 14% TDCA, 10% GDCA, 2% TCDCA and 2% GCDCA, Satiogen Pharmaceuticals), meat hydrolysate (2% w/v), 1,10-Phenanthroline (500 µm). After 2 hours of exposure, the media was collected and the resulting total PYY, and active GLP-1 concentration of the media supernatants was determined by ELISA (Cat#EZHPYYT66K, Cat#EZGLPHS-35K, EMD Millipore Sigma).

### Flow Cytometric Viability Analysis

Mouse FACS-isolated cells, as well as human FACS-isolated cells were further cultured for 24–72 h. For further viability analysis after culture, cells were recovered from Matrigel with Dispase (1 U/mL, Cat#07923, STEMCELL Technologies), and the resulting single cell suspensions were washed twice with FACS buffer and stained with PO-PRO-1 and 7-AAD. Cells were with filtered with a 30 μm cell strainer just prior to analysis. An initial 50,000 events for each unstained, and FMO controls were run in order to determine appropriate settings and gating prior to analysis. For each sample, data on 100,000 events was collected using a Fortessa X-20 flow cytometer, and cell yield and sample composition was further assessed using FlowJo.

### Statistical Analysis

Data are generally expressed as mean ± SEM unless otherwise stated. Significance testing mainly used a two-tailed parametric student t-test, unless otherwise stated. Data were analyzed in the JMP Pro (Version14) statistical software, and visualized with GraphPad Prism (Version 8). Unless otherwise stated, significance of probability values, *p < 0.05; **p < 0.01; ***p < 0.001.

## Results

### Optimization of FACS-Isolation of Cells Originating From Cryopreserved Human Mucosal Colonic Biopsies for Profiling of Intestinal Epithelial Cells With Single-Cell Transcriptomics

**FIGURE 1:**
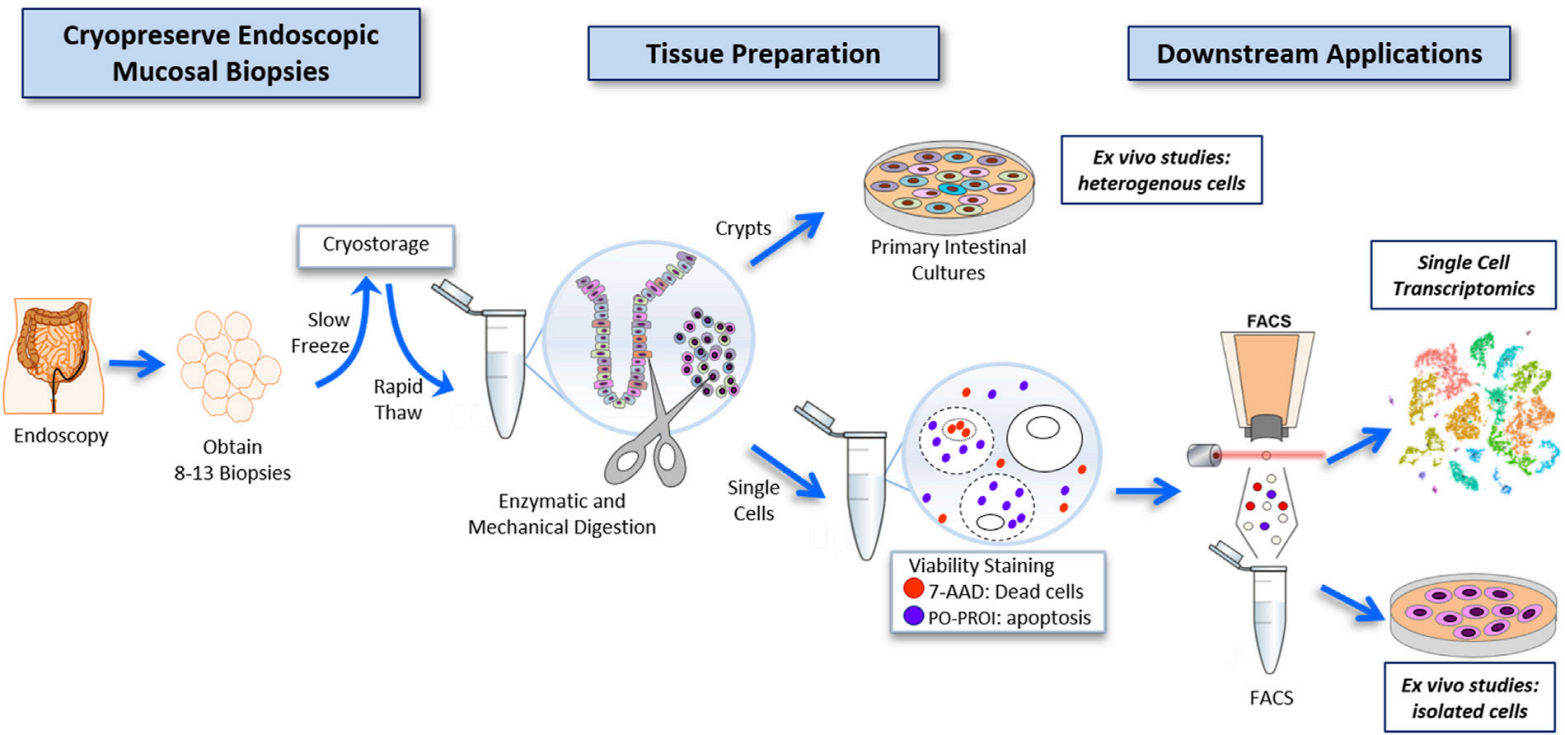
Workflow for the cryopreservation of endoscopically obtained human intestinal mucosal biopsies, and subsequent preparation of this tissue to highly viable FACS-isolated single-cell suspensions compatible with deep single-cell RNA sequencing, and primary intestinal culture.

We sought to develop an innovative protocol for the use of live human intestinal tissue, previously cryopreserved, and compatible with scRNAseq. We applied a cryopreservation protocol, originally created for human kidney tissue (Rao et al., 2018; Rao et al., 2020), and adapted its use for human mucosal biopsies of the colon. Collectively, our workflow (Figure 1) allowed for the usage of cryopreserved colon biopsies, collected from 10 participants, to be enzymatically digested to single cells, FACS-enriched, and further subjected to single-cell analysis in a single batch. Altogether, this protocol was used in downstream applications for single-cell transcriptomics, and *Ex vivo* studies of intestinal tissue with and without a FACS-enrichment component.

### FACS Gating Strategy to Exclude Low Quality Cells for Single-Cell Analysis

**FIGURE 2:**
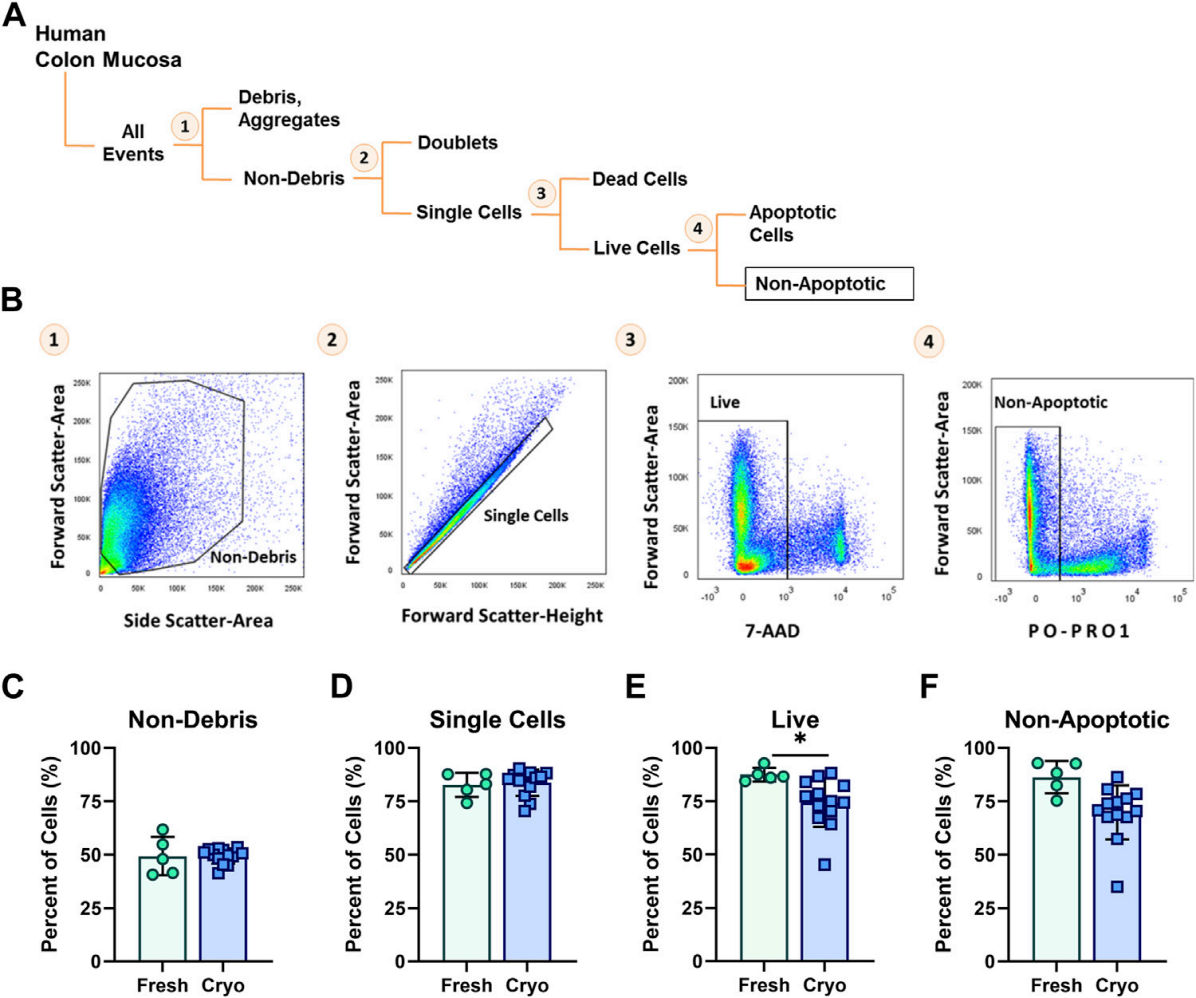
**(A)** Diagram and **(B)** corresponding FACS-plots demonstrating the sequential flow-cytometric gating strategy used to isolate cells. Average percentage of cells obtained from fresh (green circles, *n* = 5) and cryopreserved (blue squares, *n* = 13) tissue preparations throughout the population hierarchy: **(C)** Non-debris, **(D)** singlets, **(E)** Live and **(F)** Non-apoptotic cells, during FACS. Each symbol represents 1 human participant. Significance testing used a two-tailed student t-test; *p < 0.05.

Highly viable (>80%) single-cell suspensions are a critical input for successful library preparation and single-cell transcriptomics. To determine if cryopreservation of intestinal biopsies altered the morphological and basic physiological parameters of viability compared to freshly obtained tissue, we stained prepared single-cell suspensions from cryopreserved and freshly obtained tissues with viability dyes, PO-PRO-1 (early apoptosis marker) and 7-AAD (viability). The average length of time for sample storage in liquid nitrogen for cryopreserved tissue was 14 weeks, with the longest period spanning 32 weeks, and the shortest period spanning 3 weeks. Prepared cell suspensions were then analyzed using a flow cytometric gating strategy for proportions of debris, single-cells, viable, and non-apoptotic cells (Figures 2A–C). Between fresh and cryopreserved cell suspensions there were no significant differences in FACS-gating parameters for composition of non-debris and single cells (Figures 2C–D). Cell preparations originating from cryopreserved intestinal biopsies resulted in significantly decreased live cells (74.4 ± 3.2%) compared to fresh preparations (87.4 ± 1.4%) (*p* < 0.05) (Figure 2E). However, from live cells, the proportion of non-apoptotic cells was not significantly different between preparations from fresh and cryopreserved tissue (86.3 ± 3.3%, 70.0 ± 3.5%, respectively) (Figure 2F).

### Compatibility of Preparation Techniques for Single-Cell Analysis

Hypothetically, FACS-enrichment for single, live, non-apoptotic cells from suspensions originating from either cryopreserved or fresh tissue should both result in the submission of samples of purely single cells with 100% viability for scRNASeq. However the lag time to allow for all samples to be sorted per batch (4 samples per batch, 3 batches total), followed by a washing step, and delivery of isolated cells to the testing site (10 min), lead to a total wait time of 1 h from the time the first sample was sorted, to when all isolated samples were submitted to the Mayo Clinic Genome Analysis Core where the samples are subjected to the final QC viability checkpoint to determine if a sample will progress to library preparation and sequencing. The general threshold for acceptable cell viability for single-cell transcriptomics platforms is 80%, and samples must be presented as single-cell suspensions.

**FIGURE 3:**
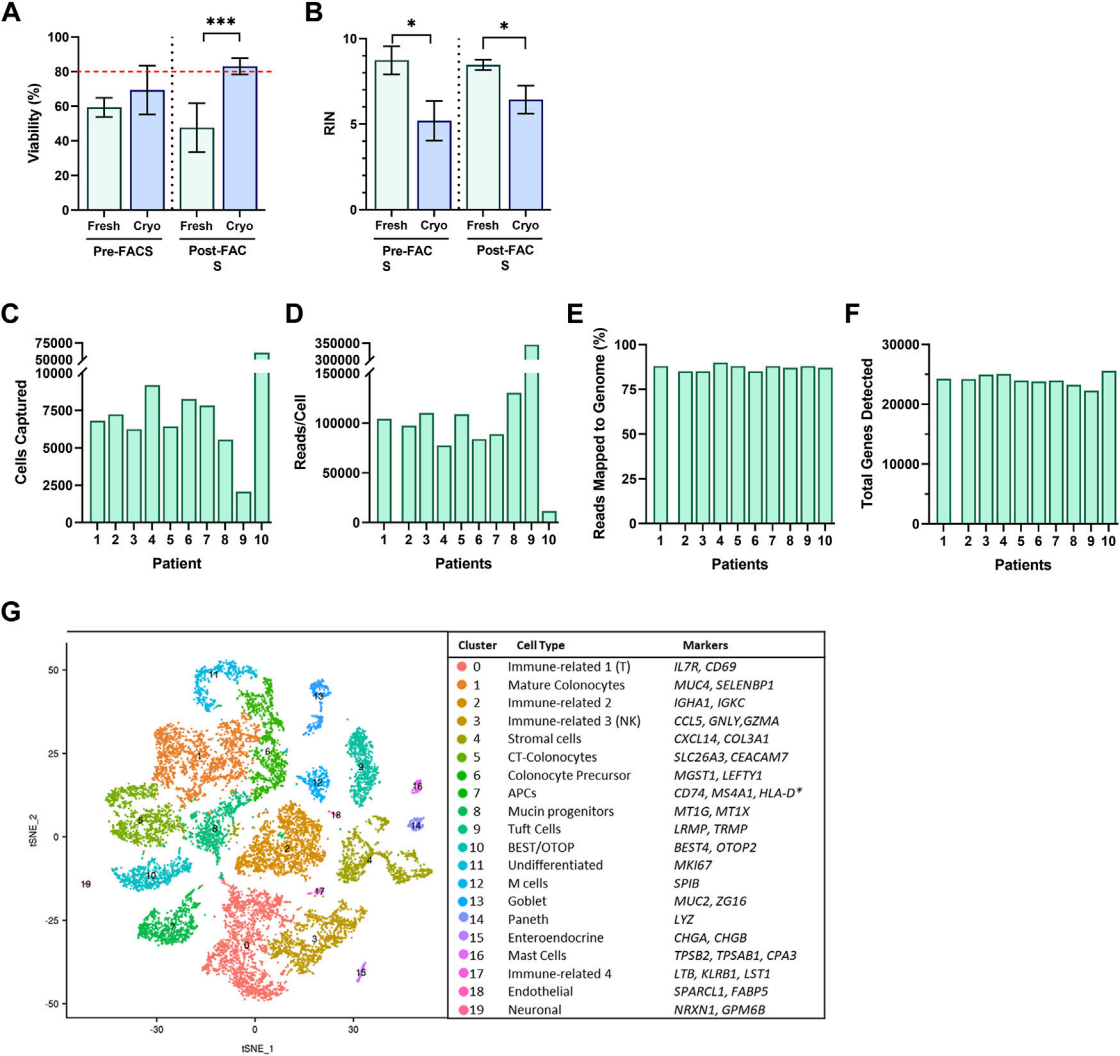
Single cells suspensions were assessed for **(A)** cell viability and **(B)** RNA Integrity Number (RIN) from fresh (green; *n* = 3, 2, for pre/post respectively) and cryopreserved (blue; *n* = 3, 10, for pre/post respectively) tissue preparations during Pre-FACS isolation and 1-h Post-FACS isolation. Summary of Data Generation from scRNA-Sequencing of FACS-isolated Human Colonic Epithelial Cells Originating from Cryopreserved Biopsies of 10 participants for **(C)** cell captured, **(D)** reads/cell, **(E)** reads mapped to genome (%), and **(F)** total genes detected. **(G)** Single-Cell RNA-Seq Profiling of Human Colonic Epithelium. t-SNE plots of single-cell RNA-seq profiles of human intestinal epithelial cells, colored by cluster number and identity, listed by largest to smallest population, and annotated by cluster identity, determined by gene markers for subtypes. Significance testing used a two-tailed student t-test; *p < 0.05, ***p < 0.001.

To determine if other strategies of our workflow would also result in acceptable viability, we tested the viability 1-h post-sort of cells resulting from freshly obtained, or cryopreserved biopsies, with or without a FACS-isolation step to excluded dead, and apoptotic cells. We investigated which workflow would meet minimum requirements for moving forward to single-cell library preparation and sequencing. Surprisingly, the only workflow with acceptable viability (>80%) for single-cell analysis of our cells, was the protocol for cryopreserved biopsies with a FACS-enriched component and resulted in 45% higher viability than freshly prepared biopsies (cryopreserved, 83.1 ± 1.6%; fresh, 47.7 ± 10.0%; *p* < 0.001) (Figure 3A). We also investigated the resulting RNA quality, and observed that regardless of a FACS component, fresh biopsies yielded cells with significantly higher RIN scores than cells from cryopreserved biopsies (*p* < 0.05) (Figure 3B). The addition of a FACS-isolation step did not correlate to a higher RNA quality in either group. Cryopreserved biopsies with a FACS component yielded a mean RIN of 6.4 ± 0.47.

### Human Colonic Epithelium Clusters Into 20 Subsets

Of the four methods, we opted to use the FACS-scRNAseq with cryopreservation of samples, to profile the human gut epithelium. This method allowed for the ability to profile patient samples collected over time, yielded samples meeting viability requirements to be compatible with single-cell platforms, while maintaining acceptable RNA quality for sequencing studies.

The cryopreserved samples of the 10 participants followed the workflow described above, which was completed in three batches to minimize time cells idled in the FACS isolation step. The initial dataset revealed sequencing of 59,653 cells with exceptional depth. The sequencing detected an average of 24,000 genes, 89% mapping to the genome, and in total, 705 million reads, at 127,000 reads per cell (Figures 3C–F) (Table 1). The number of cells captured by the Chromium 10X Controller for Patient 9, and Patient 10, as well as the number of reads per cell varied significantly from the cohort. The excess number of cells captured for Patient 10 is likely explained from a loading issue, where the number of cells was erroneously loaded to the controller either due to a dilution or pipetting miscalculation. The consequences of overloading the controller past its 10,000-cell capacity was therefore evident in the severe reduction in depth of sequencing with only 11,624 reads/cell. Patient 9 posed a contrasting matter, with only 2,071 cells captured yet astonishing sequencing depth of 345,370 read/cell. While it is feasible to propose that the sequencing depth of the low number of captured cells is valid, it is also plausible that the submitted cell suspension contained clumps of more than 1 cell, which was registered as a single-cell during the generation of Gel Bead-in-Emulsion (GEMs). For both patient samples, further bioinformatics analysis was completed to ensure the resulting t-SNE plots and expression data appears compatible with the cohort. After data filtering, we analyzed the transcriptomes of an estimated 16,723 single cells of the human gut epithelium. As expected, clustering analysis of the human colon demonstrated the remarkable heterogeneity of GI epithelium and partitioned into 20 cell subsets for which we annotated with the proposed cell type based on known markers, and conserved gene expression expressed by each subset (Figure 3G). Both common prevailing IEC subtypes, such as colonocytes, as well as rarer subsets, such as EECs, were represented in the clustering analysis.

**TABLE 1:** Summary of Data Generation from scRNAseq of FACS-isolated Human Colonic Epithelial Cells Originating from Cryopreserved Biopsies.

Participant	Number of cells	Reads/Cell	Number of read (Millions)	Reads mapped to genome (%)
1	6,811	104,224	710	88
2	7,247	97,326	705	85
3	6,245	110,210	688	85
4	9,197	77,573	713	90
5	6,435	108,889	701	88
6	8,270	83,855	693	85
7	7,830	88,870	696	88
8	5,547	130,319	723	87
9	2,071	345,370	715	88
10	60,447	11,624	702	87
Mean ± SEM	12,010 ± 5,416	115,826 ± 27,392	704.6 ± 3.4	87.1 ± 0.53

### Cryopreserved Preparation Compatibility With Post-Isolation Applications

There are many downstream applications outside of NGS platforms, for which cryopreserving intestinal tissue could be useful, such as the *ex vivo* culture of primary intestinal cells. Biopsy tissue can be prepared for the immediate culture of primary intestinal cultures through the digestion of tissue into intestinal crypts; tissue may also be digested into single-cell suspensions, immunostained with subtype-specific extracellular markers, and cultured post FACS-isolation in order to study specific IEC groups.

**FIGURE 4:**
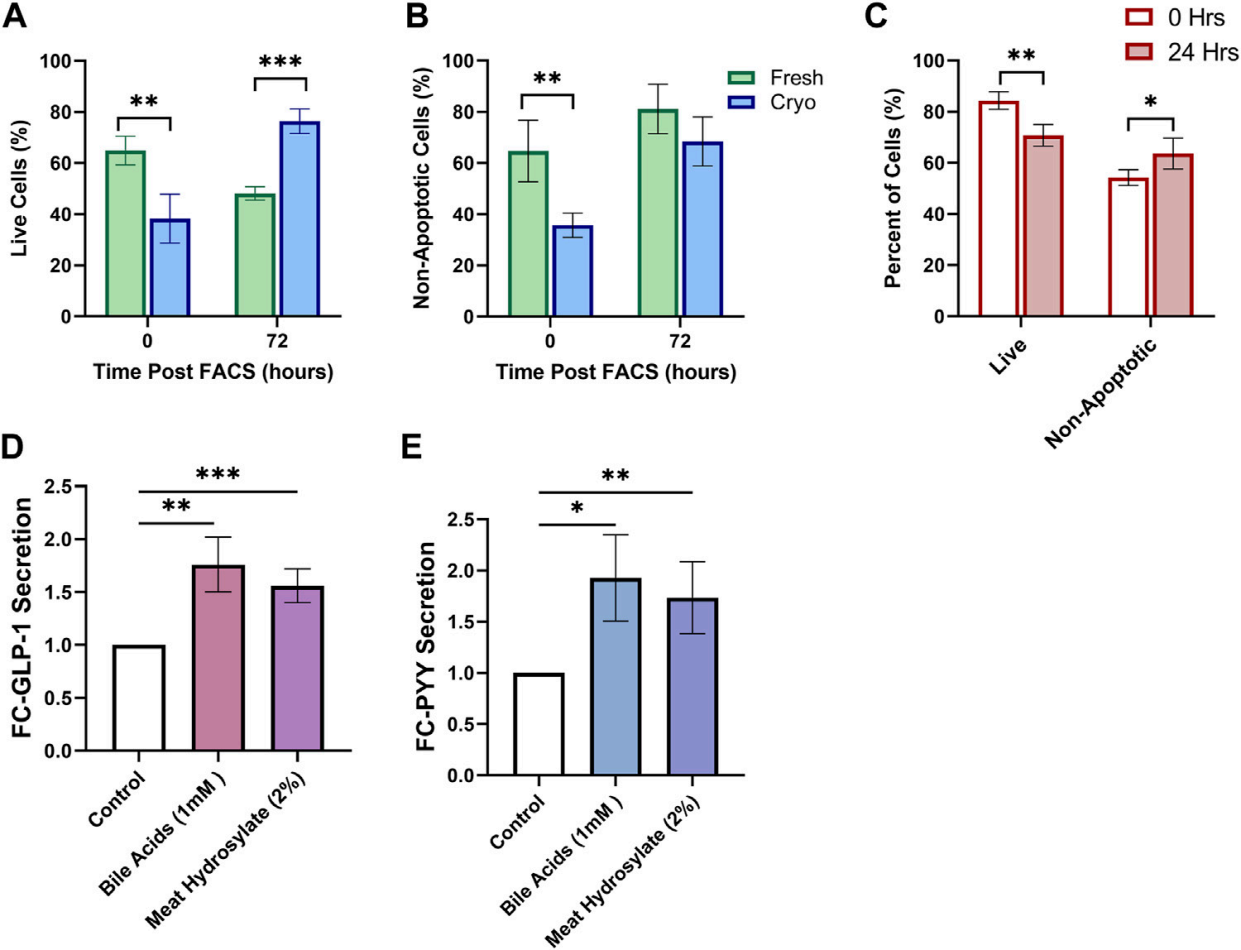
Cryopreserved Human and Mouse FACS-isolated Cells Survive in Short-term Culture. FACS-isolated cells from fresh (green, *n* = 4) or cryopreserved (blue, *n* = 4) preparations of mouse colon are analyzed by flow cytometry at initial time of sorting and 72 h posting sorting for **(A)** live cells and **(B)** non-apoptotic cell proportions after incubation in standard culture conditions. **(C)** FACS-isolated cells from cryopreserved preparations of human biopsies (*n* = 4 participants) are analyzed by flow cytometry at initial time of sorting and 24 h posting sorting for live cells and non-apoptotic cell proportions after incubation in standard culture conditions. **(D)** GLP-1 and **(E)** PYY secretion from primary intestinal cultures originating from cryopreserved human tissue (*n* = 5 replicates) treated for 2 h with PBS-0.5% BSA (control) with or without test compounds: Conjugated Bile Acids (1 mM), meat hydrolysate (2%); data expressed as normalized to protein content, and as fold-change to the control. Significance testing used a two-tailed student t-test compared between all groupings; *p < 0.05, **p < 0.01, ***p < 0.001.

Using the same tissue preparation and FACS-isolation strategy described above, we tested whether primary cultures of FACS-isolated cells from fresh and cryopreserved tissue altered survival of cultured cells in both mouse and human. Using mouse tissue, the proportion of live cells was diminished in cryopreserved preparations (38.3 ± 4.8%; *n* = 4; 3wk cryopreservation) compared to fresh (64.9 ± 2.8%; *n* = 4) (*p* < 0.01) (Figure 4A). After 72 h in culture, the viability of cells was significantly higher in cryopreserved preparations (76.4 ± 2.4%) compared to fresh (48.2 ± 1.3%) (*p* < 0.001). From the live cell fraction, the proportion of non-apoptotic cells was diminished in cryopreserved preparations (35.7 ± 2.4%) compared to fresh (64.7 ± 6.0%) (*p* < 0.01) (Figure 4B). After 72 h in culture, the proportion of non-apoptotic cells was not significantly different between preparations. Using only cryopreserved preparations from human biopsies, primary cultures of FACS-isolated cells, the proportion of live cells decreased after 24 h, whereas the proportion of live, non-apoptotic cells increased, (*p* < 0.01, *p* < 0.05, respectively) (Figure 4C).

We lastly aimed to determine if physiological assays would be successful in primary intestinal cultures originating from cryopreserved human tissue. Gut hormone secretion from EECs in response to varius nutrient stimuli can be studied from intestinal cultures; whereby intestinal tissue can be prepared for the immediate culture of primary intestinal cells through the digestion of tissue into intestinal crypts (Psichas et al., 2017). Primary human intestinal cultures appropriately responded with increased secretion of both Glucagon-like Peptide 1 (GLP-1) (Figure 4D), and Peptide-YY (PYY) (Figure 4E) when stimulated for 2-h with potent agonists, conjugated-bile acids (CBAS), and, meat hydrolysate, all known to potentiate the secretion of GLP-1 and PYY through the activation of nutrient-specific GPCRs (Brown et al., 2003; Reimer, 2006; Acosta et al., 2011; Park et al., 2015; Calderon et al., 2020).

## Discussion

The technique described in the present manuscript describes a workflow for the cryopreservation of endoscopically obtained human intestinal mucosal biopsies, and subsequent preparation of this tissue to highly viable FACS-isolated single-cell suspensions compatible with deep single-cell RNA sequencing. Cryopreserving mucosal biopsies from participants in the study not only enabled the accrual of specimens collected over time, but also permitted for all sample processing and single-cell analysis to be completed in a single batch. Furthermore, primary intestinal cultures were successfully generated from cryopreserved tissue and capable of surviving in short-term culture conditions in human and mouse and were suitable for physiological assays studying gut peptide secretion from rare hormone-producing EECs in humans.

Our ability to study IECs in many disease states in humans from native tissue has been limited due to longstanding technical and logistical challenges to their study. Using traditional methods, studies attempting to perform a transcriptomic analysis of colonic tissue need to navigate multiple challenges related to tissue collection and processing (Rao et al., 2018; Rao et al., 2020). First, clinical research sites must obtain tissue from specific patients for research analysis that is frequently obtained from surgical specimens, largely inaccessible to many research institutions. The approach described here offers compatibility with intestinal biopsy tissue, and therefore presents an advantageous opportunity for greater selectivity of study participants undergoing more routine and relatively noninvasive procedures, like flexible sigmoidoscopies, used in the study, or from upper endoscopies or colonoscopies. This departs from traditional processes of obtaining human intestinal specimens for NGS platforms, which commonly rely on collecting tissue resections from patients undergoing surgical procedures.

After collection, tissue samples need to be immediately processed to obtain viable single-cell suspensions for scRNAseq analysis as rapid processing minimizes transcriptional changes in gene expression and current scRNAseq methods require highly viable single cells for analysis (Saliba et al., 2014). Multiple technical variables throughout processing, including handling and incubation times, temperatures, and dissociation conditions can alter the transcriptomes of isolated cells. For this reason, intricate processing logistics also render samples prepared in multiple batches problematic. Therefore, optimization of the processing of tissue samples represents a critical step dictating quality of transcriptomic data. Here we address the major limitation of batch-to-batch technical variation (Leek et al., 2010; Lahnemann et al., 2020) by cryopreserving endoscopic mucosal biopsy samples as intact tissue upon collection using methods similar to those used to cryopreserve viable cells. Cryopreserving intact tissue samples offers several advantages: it minimizes the handling required at study sites as tissue can be rapidly frozen after acquisition; it enables the accumulation of a biobank of intact tissue that can be sent to a central processing site, and viable upon thawing; and it allows the accumulation of samples at a central processing site, where they can be processed and analyzed in large batches to reduce batch-to-batch technical variation.

The method described in this study addresses many of current limitations to conducting scRNASeq in humans (Saliba et al., 2014), and may allow for the expansion of scRNASeq compatible studies with larger numbers of participants to be involved in studies, for studies to be conducted with interventions, at multiple sites. Furthermore, outcomes and observations from these studies could offer greater translation to human disease as native tissue is directly obtained from its original environment and may therefore allow for greater preservation of pathophysiological signatures.

This study has some limitations that are necessary to be addressed in the future. First, in the present manuscript we do not offer an assessment of the single-cell transcriptomic landscape between resulting preparations of fresh and cryopreserved tissue. While our workflow adopts a FACS-isolation component to secure that cells from cryopreserved preparations include only highly viable single cells for downstream analysis, we cannot indubitably conclude that the transcriptomes between these fresh and cryopreserved cells would be devoid of relevant transcriptomic alterations due to the cryopreservation process. However previous studies using an approach akin to the protocol reported here, but omitting the FAC-isolation step to exclude poor quality cells, still reported minimal (<5%) transcriptomic alterations in transcriptomes of preserved tissue compared to fresh tissue in kidney (Rao et al., 2018; Rao et al., 2020). These studies offer some promise to the transcriptomic integrity of cells from cryopreserved tissue for single-cell analyses. Yet nonetheless, additional studies comparing transcriptomic profiles between fresh and cryopreserved tissue using human intestinal tissue with the workflow presented in this manuscript are necessary. Also, we did not investigate whether the length of time a tissue is cryopreserved alters viability parameters. This information would be crucial in order to extend this protocol to biobanking efforts where tissues may need to be stored over the course of years. Finally, this study only investigated the suitability of primary intestinal cultures for physiological assays studying gut peptide secretion from EECs in humans. The compatibility of this method for the study of other IEC subtypes and their various physiological functions in primary culture requires additional validation.

In summary, this workflow can be applied to live human intestinal tissue, from colonic mucosal biopsies, previously cryopreserved, compatible with scRNAseq. The presented workflow provides a more accessible avenue for researchers to conduct NGS level studies to directly evaluate distinct population present in the gut mucosa.

## Data Availability

The datasets presented in this study can be found in online repositories. The names of the repository/repositories and accession number(s) can be found below: https://www.ncbi.nlm.nih.gov/, GSE154405.
